# Phenethyl isothiocyanate and paclitaxel synergistically enhanced apoptosis and alpha-tubulin hyperacetylation in breast cancer cells

**DOI:** 10.1186/2162-3619-3-5

**Published:** 2014-02-05

**Authors:** Shundong Cang, Yuehua Ma, Jen-wei Chiao, Delong Liu

**Affiliations:** 1 Department of Oncology, Henan Province People’s Hospital, Zhengzhou University, Zhengzhou, China; 2 Department of Medicine, New York Medical College and Westchester Medical Center, Valhalla, NY 10595, USA; 3 Institute of Hematology, Henan Tumor Hospital, Zhengzhou University, Zhengzhou, China

## Abstract

Combination of phenethyl isothiocyanate (PEITC) and paclitaxel (taxol) has been shown to work synergistically to increase apoptosis and cell cycle arrest in breast cancer cells. In this report, we further explored the mechanisms for the synergistic activity of PEITC and taxol in MCF7 and MDA-MB-231 (MB) breast cancer cell lines. By Western blotting analysis, treatment of MCF7 cells with both PEITC and taxol led to a 10.4-fold and 5.96-fold increase in specific acetylation of alpha-tubulin over single agent PEITC and taxol, respectively. This synergistic effect on acetylation of alpha-tubulin was also seen in MB cells. The combination of PEITC and taxol also reduced expressions of cell cycle regulator Cdk1, and anti-apoptotic protein bcl-2, enhanced expression of Bax and cleavage of PARP proteins. In conclusion, this study provided biochemical evidence for the mechanism of synergistic effect between the epigenetic agent PEITC and the chemotherapeutic agent taxol.

## Introduction

Epigenetic modification of DNA and histone proteins by methylation and deacetylation plays a key role in carcinogenesis [1-5]. Methyltransferase inhibitors and histone deacetylase (HDAC) inhibitors are novel anti-cancer agents. Two DNA methyltransferase inhibitors, azacitidine and decitabine, and two histone deacetylase inhibitors, vorinostat and romidepsin, have been in clinical use [6-12]. Belinostat was reported to induce durable remission in refractory peripheral T-cell lymphoma [13].

Breast cancer is the most commonly diagnosed cancer and the second leading cause of death among women. Taxanes are a class of major chemotherapeutic agents for breast cancer therapy. Paclitaxel (taxol) is a widely used chemotherapy drug in the treatment of breast cancer and other solid tumors [14-16]. Taxol inhibits microtubule disassembly when it binds to assembled tubulin, making the microtubules locked in polymerized state [17]. Thus the taxol -exposed cells are in cell cycle arrest [18-21]. Another effect of taxol is that it inhibits the anti-apoptosis protein Bcl-2, and induces apoptosis in cancer cells [22]. Even though taxol is a highly effective anti-neoplastic agent, the toxicity of taxol, particularly at a higher dosage, limits its prolonged use in patients [15,23,24]. Further research is being done to increase therapeutic efficacy and minimize toxicity. Radiation and targeted therapy has been used effectively for breast cancer therapy [25-28]. Novel anti-cancer agents with novel mechanisms of actions and new formulations are being actively sought [29-31].

Phenethyl isothiocyanate (PEITC) belongs to the family of isothiocyanates, which are initially found in a wide variety of cruciferous vegetables. Natural ITCs are released when the vegetables are cut or masticated. Phenethyl isothiocyanate (PEITC) regulates epigenetic process. PEITC has been shown to be a HDAC inhibitor in prostate cancer, leukemia, and myeloma cells [32-35]. PEITC was also shown to inhibit leukemia development in mice. PEITC was shown to have dual functions and can induce DNA hypomethylation as well as histone hyperacetylation [34,36]. Our group has recently shown that combination of PEITC and taxol has synergistic inhibitory effects on breast cancer cell growth [37]. The combination synergistically increased apoptosis and cell cycle arrest in breast cancer cells. In this report, we further explored the mechanisms for the synergistic activity of PEITC and taxol.

## Materials and methods

### Chemicals and cell cultures

As described in previous reports [33-35], PEITC (phenethyl isothiocyanate) was purchased from LKT Labs and dissolved in 70% methanol and 30% deionized water to a stock concentration of 10 mM. Paclitaxel (taxol) powder (Sigma Chemical Co.) was dissolved in DMSO and stored as a stock concentration of 200 nM.

Maintenance and culture of the MCF7 and MDA-MB-231 (MB) cell lines were described in a prior report [37]. Briefly, the cells were seeded at 0.4 × 10^6^ per ml and 0.2 × 10^6^ per ml, respectively, of PRMI-1640 medium supplemented with 10% heat-inactivated fetal bovine serum, 100 IU of penicillin/ml and 100 μg of streptomycin/ml, and maintained at 37°C in a humidified atmosphere containing 5% CO_2_. At the specified time points, the cells were harvested. Cell number and viability were determined from at least triplicate cultures by the trypan blue exclusion method.

### Western blotting

MCF and MB cells were treated with PEITC and/or paclitaxel at various concentrations for 48 hours. The cell lysates were used for Western blot analysis as described previously [38,39]. The protein content of the lysates was determined using the BioRad Protein Assay Kit (BioRad, Hercules, CA), with a BSA standard. The antibodies against the following proteins were used for immunoblotting: PARP-1, BCL-2, Bax, Cdk-1, Cyclin B1, α-tubulin, β-tubulin, β-actin, acetyl-α-tubulin, HDAC6, acetyl-H3, and Acetyl-H4 [34,35,40]. Secondary antibodies were chosen according to the primary antibodies used (goat anti-rabbit or anti- mouse IgG antibody linked to HRP, Santa Cruz). The proteins were visualized through the ECL system. The protein was quantified using the β-actin protein as the loading control.

### Confocal immunofluorescence

Immunostaining of cells for confocal immunofluorescence microscopy was done according to the published methods [35]. Briefly the MCF and MB cells grown on chamber slides were treated for 48 hours without or with PEITC, the cells were then fixed, permeabilized, blocked in BSA and incubated with a mouse anti-acetyl-α-tubulin (Sigma-Aldrich) for 1 h. A fluorescin-conjugated goat anti-mouse IgG was used as secondary antibody. The DNA was counterstained with propidium iodide (PI) to visualize the nuclei of the cells. Images were captured using an MRC 1024 ES confocal laser scanning microscopy system.

## Results

### PEITC and taxol increased acetylation of alpha-tubulin in breast cancer cells

**Figure 1:**
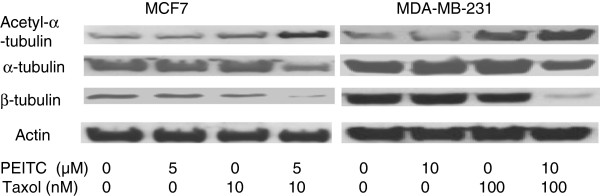
**Synergistic effect of phenethyl isothiocyanate (PEITC) and paclitaxel (taxol) on acetylation of alpha-tubulin and expression of tubulins in breast cancer cells.** The MCF7 cells were treated with 5 μM of PEITC and 10 nM of taxol alone or in combination. Due to higher resistance of MDA-MB-231 cells, PEITC at 10 μM and taxol at 100 nM were used. Proteins were immunoprecipitated with appropriate antibodies and were detected by Western blotting as described in Materials and methods. β-actin level was used as an internal loading control for protein amount. The relative expression level over control cells was calculated after scanning of the density of each protein band with a densitometer. A representative blot from three or more independent experiments was shown.

Alpha-tubulin has been shown to be acetylated by HDAC6 [41]. When the cells were treated with the combination of PEITC and taxol, the acetylation of alpha-tubulin was significantly increased in both MCF and MB cells in comparison with that in single agent treated cells (Figure 1). When the acetylation level was corrected for the amount of total alpha-tubulin present in the specimen, there was a 16% and 28% respective increase in the specific acetylation level (SAL) of acetylated alpha-tubulin (acetyl-alpha-tubulin per unit of total alpha-tubulin) in MCF cells treated with PEITC or taxol alone (Figure 1). There was a 167% increase in SAL in MCF cells treated with both PEITC and taxol. Therefore, the combination led to a 10.4-fold and 5.96-fold increase in SAL over single agent PEITC and taxol, respectively. This synergistic effect on acetylation of alpha-tubulin was also seen in MB cells (Figure 1). Interestingly, taxol alone also enhanced acetylation of alpha-tubulin in both cell lines. The combination also decreased expression of beta-tubulin more than each agent alone.

**Figure 2:**
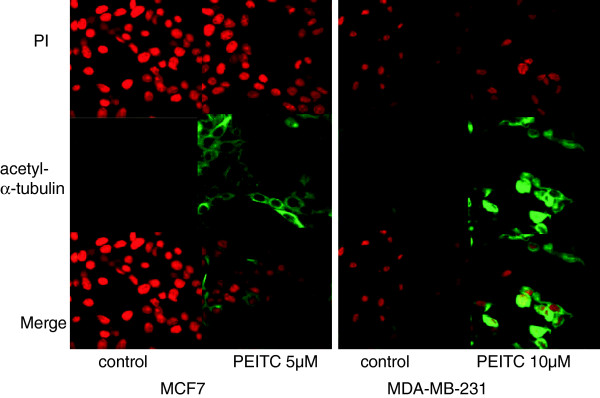
**Acetyl-alpha-tubulin immunofluorescence in the cytoplasm of MCF7 and MDA-MB-231 breast cancer cells.** The MCF7 cells and MDA-MB-231 cells were treated with or without PEITC. The cells were stained with anti-acetyl-alpha-tubulin. Fluorescin-labeled secondary antibody was then added. The cells were visualized under confocal fluorescent microscope. The nuclei were counter stained with propidium iodide (PI). In the treated cells, acetylated alpha-tubulin was seen in the cytoplasm.

To directly visualize the activity of PEITC on breast cancer cells in live cell culture, we next studied the level and distribution of acetylated alpha-tubulin by immunostaining. The cells were visualized with confocal fluorescent microscopy. The cytoplasmic level of acetylated alpha-tubulin clearly increased in both MCF and MB cells after treatment with 5 μM of PEITC for 48 hours, which can be directly visualized under confocal fluorescent microscope (Figure 2).

### Effect of combination of PEITC and taxol on cyclin B1 and CDK1 expression

**Figure 3:**
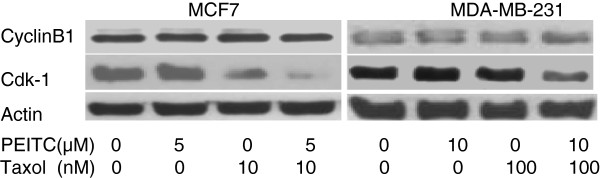
**Combination of phenethyl isothiocyanate (PEITC) and paclitaxel (taxol) inhibits cell cycle regulator cyclin B1 and Cdk1 in MCF7 and MDA-MB-231 breast cancer cells.** The MCF7 cells were treated with 5 μM of PEI and 10 nM of taxol alone or in combination. Due to higher resistance of MDA-MB-231 cells, PEI at 10 μM and taxol at 100 nM were used. Cyclin B1 and Cdk1 were immunoprecipitated with appropriate antibodies and were detected by Western blotting as described in Materials and methods. β-actin level was used as an internal loading control for protein amount. A representative blot from three or more independent experiments was shown.

Cyclin B1 and CDK1 are major cell cycle regulatory proteins for the G2 to M phase progression [42]. To explore the involvement of the major cell cycle regulatory proteins, the level of cyclin B1 and CDK1 expression was studied. Their expressions were characterized with Western blotting. When compared with single agent PEITC and taxol, the combination of both agents reduced the expression of CDK1 more significantly than either agent alone (Figure 3). In the mean time, the cyclin B1 expression was minimally decreased, indicating a less significant effect from the treatment.

### Effect of combination of PEITC and taxol on Bax and Bcl-2 expression

**Figure 4:**
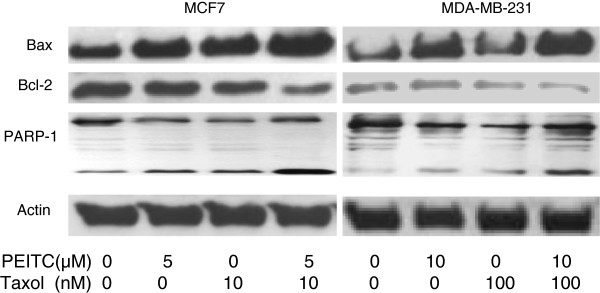
**Synergistic effect of phenethyl isothiocyanate (PEITC) and paclitaxel (taxol) on expressions of Bcl-2, Bax, PARP in MCF7 and MDA-MB-231 breast cancer cells.** The MCF7 cells were treated with 5 μM of PEITC and 10 nM of taxol alone or in combination. Due to higher resistance of MDA-MB-231 cells, PEITC at 10 μM and taxol at 100 nM were used. Cell lysates were immunoprecipitated with appropriate antibodies and were detected by Western blotting as described in Materials and methods. β-actin level was used as an internal loading control for protein amount. A representative blot from three or more independent experiments was shown.

Bax and Bcl-2 have opposing effects on apoptosis. Bax promotes apoptosis while Bcl-2 is an anti-apoptosis protein. The levels of the two proteins in the breast cancer cell lines were examined through Western blotting analysis. When compared with single agent PEITC and taxol, the combination of both agents reduced Bcl-2 expression and increased Bax expression more than either agent alone (Figure 4).

### Effect of combination of PEITC and taxol on PARP cleavage

PARP proteins are important downstream components of the apoptosis pathways. Cell cycle arrest usually triggers the apoptosis machinery which leads to cellular apoptosis and cell death. The PARP protein cleavage in MCF and MB cells was examined. When compared with single agent PEITC and taxol, the combination of both agents increased the PARP-1 cleavage (thus an increase in the degradation products as described [43]) more than either agent alone in both cell lines (Figure 4).

## Discussion

It has been shown that tubulin acetylation primarily occurs on assembled microtubules [44]. PEITC has been previously found to directly bind to alpha- and beta- tubulins, thus inhibiting microtubule polymerization in prostate cancer cells [45]. In this study, PEITC was shown, for the first time, to induce hyperacetylation of alpha-tubulin in two different breast cancer cell lines. It is possible that PEITC can inhibit the synthesis of alpha-tubulin deacetylase HDAC6 [41] (data not shown). This may help to explain the previous findings that some HDAC inhibitors, such as TSA but not butyric acid, can cause alpha-tubulin hyperacetylation [46]. This study also provided evidence to illustrate the possible mechanisms for the synergistic anti-growth effect of PEITC and taxol to be due to hyperacetylation of alpha-tubulin. This synergism is best explained by the fact that taxol enhances tubulin acetylation by inhibiting depolymerization of microtubules and thus leads to availability of more substrates for acetylases, whereas PEITC decreases tubulin deacetylation.

This study also showed that the combination of PEITC and taxol enhanced apoptosis by decreasing bcl-2 expression and by increasing BAX expression as well as degradation of PARP. The combination of the two agents also reduced CDK1 expression. These biochemical data provided the foundation of the mechanisms for the synergistic effects of the two agents on apoptosis and cell cycle arrest. The similar mechanism was also found to be responsible for PEITC inhibition of prostate cancer cells [32,35,47-49]. Further study of this effect on prostate cancer cells are ongoing in our laboratory.

Our lab and others have shown that PEITC has little toxic effects on normal cells [35,38,50]. However, taxol has significant toxicity at higher dosage and after prolonged use. We therefore hypothesize that by combining PEITC and taxol, it is possible to significantly reduce toxicity *in vivo* by reducing the dosage of taxol needed while maintaining clinical efficacy for breast cancer and possibly other solid tumors. This hypothesis will be tested first in mouse model carrying breast cancer xenografts.

The HDAC inhibitor vorinostat has been shown to up-regulate estrogen receptors and make breast cancer cells more sensitive to tamoxifen [51]. HDAC inhibitor was found to redirect the response of breast cancers cells to tamoxifen from cell cycle arrest to apoptosis [52]. Since PEITC is a HDAC inhibitor as well as a tubulin-targeting agent, it would be worthwhile to test the combination of PEITC and tamoxifen for therapy of hormone-refractory breast cancer.

## Conclusion

This study provided biochemical evidence for the mechanism of synergistic effect between the epigenetic agent PEITC and the chemotherapeutic agent taxol. This novel strategy deserves further study *in vivo* in animal models and may provide a new and enhanced treatment option for breast cancer patients.

## Competing interests

The authors have no relevant competing interests.

## Authors’ contributions

All authors have contributed to data preparation, drafting and revising the manuscripts. All authors have read and approved the final manuscript.

## References

[B1] Wolffe AP Matzke MA Epigenetics: regulation through repression Science 1999 286 5439 481 486 10.1126/science.286.5439.481 10521337

[B2] Workman JL Kingston RE Alteration of nucleosome structure as a mechanism of transcriptional regulation Annu Rev Biochem 1998 67 545 579 10.1146/annurev.biochem.67.1.545 9759497

[B3] Ura K Kurumizaka H Dimitrov S Almouzni G Wolffe AP Histone acetylation: influence on transcription, nucleosome mobility and positioning, and linker histone-dependent transcriptional repression EMBO J 1997 16 8 2096 2107 10.1093/emboj/16.8.2096 9155035 PMC1169812

[B4] Bird AP Wolffe AP Methylation-induced repression–belts, braces, and chromatin Cell 1999 99 5 451 454 10.1016/S0092-8674(00)81532-9 10589672

[B5] Gutierrez S Romero-Oliva F Epigenetic changes: a common theme in acute myelogenous leukemogenesis J Hematol Oncol 2013 6 1 57 10.1186/1756-8722-6-57 23938080 PMC3751780

[B6] Chakraborty AR Robey RW Luchenko VL Zhan Z Piekarz RL Gillet JP Kossenkov AV Wilkerson J Showe LC Gottesman MM MAPK pathway activation leads to Bim loss and histone deacetylase inhibitor resistance: rationale to combine romidepsin with a MEK inhibitor Blood 2013 121 20 4115 4125 10.1182/blood-2012-08-449140 23532732 PMC3656450

[B7] Sherman EJ Su YB Lyall A Schoder H Fury MG Ghossein RA Haque S Lisa D Shaha AR Tuttle RM Evaluation of romidepsin for clinical activity and radioactive iodine reuptake in radioactive iodine-refractory thyroid carcinoma Thyroid 2013 23 5 593 599 10.1089/thy.2012.0393 23186033 PMC3643228

[B8] Pleyer L Stauder R Burgstaller S Schreder M Tinchon C Pfeilstocker M Steinkirchner S Melchardt T Mitrovic M Girschikofsky M Azacitidine in patients with WHO-defined AML – results of 155 patients from the Austrian Azacitidine Registry of the AGMT-Study Group J Hematol Oncol 2013 6 1 32 10.1186/1756-8722-6-32 23627920 PMC3655844

[B9] van der Helm L Scheepers E Veeger N Daenen S Mulder A van den Berg E Vellenga E Huls G Azacitidine might be beneficial in a subgroup of older AML patients compared to intensive chemotherapy: a single centre retrospective study of 227 consecutive patients J Hematol Oncol 2013 6 1 29 10.1186/1756-8722-6-29 23587459 PMC3639930

[B10] Gojo I Tan M Fang HB Sadowska M Lapidus R Baer MR Carrier F Beumer JH Anyang BN Srivastava RK Translational phase I trial of vorinostat (Suberoylanilide Hydroxamic Acid) combined with cytarabine and etoposide in patients with relapsed, refractory, or high-risk acute myeloid leukemia Clin Cancer Res 2013 19 7 1838 1851 10.1158/1078-0432.CCR-12-3165 23403629 PMC4332848

[B11] Xu S De Veirman K Evans H Santini GC Vande Broek I Leleu X De Becker A Van Camp B Croucher P Vanderkerken K Effect of the HDAC inhibitor vorinostat on the osteogenic differentiation of mesenchymal stem cells in vitro and bone formation in vivo Acta Pharmacol Sin 2013 34 5 699 709 10.1038/aps.2012.182 23564084 PMC4002867

[B12] Fianchi L Criscuolo M Lunghi M Gaidano G Breccia M Levis A Finelli C Santini V Musto P Oliva E Outcome of therapy-related myeloid neoplasms treated with azacitidine J Hematol Oncol 2012 5 1 44 10.1186/1756-8722-5-44 22853048 PMC3419605

[B13] Reimer P Chawla S Long-term complete remission with belinostat in a patient with chemotherapy refractory peripheral t-cell lymphoma J Hematol Oncol 2013 6 1 69 10.1186/1756-8722-6-69 24020452 PMC3846498

[B14] Holmes FA Walters RS Theriault RL Forman AD Newton LK Raber MN Buzdar AU Frye DK Hortobagyi GN Phase II trial of taxol, an active drug in the treatment of metastatic breast cancer J Natl Cancer Inst 1991 83 24 1797 1805 1683908 10.1093/jnci/83.24.1797-a

[B15] Brown T Havlin K Weiss G Cagnola J Koeller J Kuhn J Rizzo J Craig J Phillips J Von HD A phase I trial of taxol given by a 6-hour intravenous infusion J Clin Oncol 1991 9 7 1261 1267 1675263 10.1200/JCO.1991.9.7.1261

[B16] McGuire WP Rowinsky EK Rosenshein NB Grumbine FC Ettinger DS Armstrong DK Donehower RC Taxol: a unique antineoplastic agent with significant activity in advanced ovarian epithelial neoplasms Ann Intern Med 1989 111 4 273 279 10.7326/0003-4819-111-4-273 2569287

[B17] Jordan MA Kamath K How do microtubule-targeted drugs work? An overview Curr Cancer Drug Targets 2007 7 8 730 742 10.2174/156800907783220417 18220533

[B18] Fuchs DA Johnson RK Cytologic evidence that taxol, an antineoplastic agent from Taxus brevifolia, acts as a mitotic spindle poison Cancer TreatRep 1978 62 8 1219 1222 688258

[B19] Schiff PB Horwitz SB Taxol stabilizes microtubules in mouse fibroblast cells Proc Natl Acad Sci USA 1980 77 3 1561 1565 10.1073/pnas.77.3.1561 6103535 PMC348536

[B20] Schiff PB Horwitz SB Taxol assembles tubulin in the absence of exogenous guanosine 5′-triphosphate or microtubule-associated proteins Biochemistry 1981 20 11 3247 3252 10.1021/bi00514a041 6113842

[B21] Schiff PB Fant J Horwitz SB Promotion of microtubule assembly in vitro by taxol Nature 1979 277 5698 665 667 10.1038/277665a0 423966

[B22] Haldar S Jena N Croce CM Inactivation of Bcl-2 by phosphorylation Proc Natl Acad Sci USA 1995 92 10 4507 4511 10.1073/pnas.92.10.4507 7753834 PMC41973

[B23] Wiernik PH Schwartz EL Strauman JJ Dutcher JP Lipton RB Paietta E Phase I clinical and pharmacokinetic study of taxol Cancer Res 1987 47 9 2486 2493 2882837

[B24] Wiernik PH Schwartz EL Einzig A Strauman JJ Lipton RB Dutcher JP Phase I trial of taxol given as a 24-hour infusion every 21 days: responses observed in metastatic melanoma J Clin Oncol 1987 5 8 1232 1239 2887641 10.1200/JCO.1987.5.8.1232

[B25] Olivotto IA Whelan TJ Parpia S Kim D-H Berrang T Truong PT Kong I Cochrane B Nichol A Roy I Interim cosmetic and toxicity results from RAPID: a randomized trial of accelerated partial breast irradiation using three-dimensional conformal external beam radiation therapy J Clin Oncol 2013 31 doi:10.1200/JCO.2013.1250.5511 10.1200/JCO.2013.50.5511 23835717

[B26] Chiang H-C Nair S Yeh I-T Santillan A Hu Y Elledge R Li R Association of radiotherapy with preferential depletion of luminal epithelial cells in a BRCA1 mutation carrier Exp Hematol Oncol 2012 1 1 31 10.1186/2162-3619-1-31 23210696 PMC3514114

[B27] Incorvati J Shah S Mu Y Lu J Targeted therapy for HER2 positive breast cancer J Hematol Oncol 2013 6 1 38 10.1186/1756-8722-6-38 23731980 PMC3703272

[B28] Braccini AL Azria D Thezenas S Romieu G Ferrero JM Jacot W Prognostic factors of brain metastases from breast cancer: impact of targeted therapies Breastdoi:10.1016/j.breast.2013.1005.1011 10.1016/j.breast.2013.05.011 23831232

[B29] Rafiyath S Rasul M Lee B Wei G Lamba G Liu D Comparison of safety and toxicity of liposomal doxorubicin vs. conventional anthracyclines: a meta-analysis J Hematol Oncol 2012 1 1 10 10.1186/2162-3619-1-10 PMC3514106 23210520

[B30] Elbaz H Stueckle T Tse W Rojanasakul Y Dinu C Digitoxin and its analogs as novel cancer therapeutics Exp Hematol Oncol 2012 1 1 4 10.1186/2162-3619-1-4 23210930 PMC3506989

[B31] Xu D Wang Q Jiang Y Zhang Y Vega-SaenzdeMiera E Osman I Dai W Roles of Polo-like kinase 3 in suppressing tumor angiogenesis Exp Hematol Oncol 2012 1 1 5 10.1186/2162-3619-1-5 23210979 PMC3506990

[B32] Beklemisheva AA Fang Y Feng J Ma X Dai W Chiao JW Epigenetic mechanism of growth inhibition induced by phenylhexyl isothiocyanate in prostate cancer cells Anticancer Res 2006 26 2A 1225 1230 16619528

[B33] Wang LG Liu XM Fang Y Dai W Chiao FB Puccio GM Feng J Liu D Chiao JW De-repression of the p21 promoter in prostate cancer cells by an isothiocyanate via inhibition of HDACs and c-Myc Int J Oncol 2008 33 2 375 380 18636159

[B34] Wang LG Beklemisheva A Liu XM Ferrari AC Feng J Chiao JW Dual action on promoter demethylation and chromatin by an isothiocyanate restored GSTP1 silenced in prostate cancer Mol Carcinog 2007 46 1 24 31 10.1002/mc.20258 16921492

[B35] Cang S Feng J Konno S Han L Liu K Sharma SC Choudhury M Chiao JW Deficient histone acetylation and excessive deacetylase activity as epigenomic marks of prostate cancer cells Int J Oncol 2009 35 1417 1422 19885564 10.3892/ijo_00000459

[B36] Zou Y Ma X Huang Y Hong L Chiao J-w Effect of phenylhexyl isothiocyanate on aberrant histone H3 methylation in primary human acute leukemia J Hematol Oncol 2012 5 1 36 10.1186/1756-8722-5-36 22747680 PMC3413588

[B37] Liu K Cang S Ma Y Chiao JW Synergistic effect of paclitaxel and epigenetic agent phenethyl isothiocyanate on growth inhibition, cell cycle arrest and apoptosis in breast cancer cells Cancer Cell Int 2013 13 1 10 10.1186/1475-2867-13-10 23388416 PMC3637186

[B38] Ma X Fang Y Beklemisheva A Dai W Feng J Ahmed T Liu D Chiao JW Phenylhexyl isothiocyanate inhibits histone deacetylases and remodels chromatins to induce growth arrest in human leukemia cells Int J Oncol 2006 28 5 1287 1293 16596246

[B39] Wade PA Transcriptional control at regulatory checkpoints by histone deacetylases: molecular connections between cancer and chromatin Hum Mol Genet 2001 10 7 693 698 10.1093/hmg/10.7.693 11257101

[B40] Lu Q Lin X Feng J Zhao X Gallagher R Lee MY Chiao JW Liu D Phenylhexyl isothiocyanate has dual function as histone deacetylase inhibitor and hypomethylating agent and can inhibit myeloma cell growth by targeting critical pathways J Hematol Oncol 2008 1 6 10.1186/1756-8722-1-6 18577263 PMC2438442

[B41] Hubbert C Guardiola A Shao R Kawaguchi Y Ito A Nixon A Yoshida M Wang XF Yao TP HDAC6 is a microtubule-associated deacetylase Nature 2002 417 6887 455 458 10.1038/417455a 12024216

[B42] Lindqvist A van ZW Karlsson RC Wolthuis RM Cyclin B1-Cdk1 activation continues after centrosome separation to control mitotic progression PLoS Biol 2007 5 5 e123 10.1371/journal.pbio.0050123 17472438 PMC1858714

[B43] Chaitanya G Alexander J Babu P PARP-1 cleavage fragments: signatures of cell-death proteases in neurodegeneration Cell Commun Signal 2010 8 1 31 10.1186/1478-811X-8-31 21176168 PMC3022541

[B44] Piperno G LeDizet M Chang XJ Microtubules containing acetylated alpha-tubulin in mammalian cells in culture J Cell Biol 1987 104 2 289 302 10.1083/jcb.104.2.289 2879846 PMC2114420

[B45] Mi L Xiao Z Hood BL Dakshanamurthy S Wang X Govind S Conrads TP Veenstra TD Chung FL Covalent binding to tubulin by isothiocyanates. A mechanism of cell growth arrest and apoptosis J Biol Chem 2008 283 32 22136 22146 10.1074/jbc.M802330200 18524779 PMC2494917

[B46] Dowdy SC Jiang S Zhou XC Hou X Jin F Podratz KC Jiang SW Histone deacetylase inhibitors and paclitaxel cause synergistic effects on apoptosis and microtubule stabilization in papillary serous endometrial cancer cells Mol Cancer Ther 2006 5 11 2767 2776 10.1158/1535-7163.MCT-06-0209 17121923

[B47] Xiao D Powolny AA Moura MB Kelley EE Bommareddy A Kim SH Hahm ER Normolle D Van Houten B Singh SV Phenethyl isothiocyanate inhibits oxidative phosphorylation to trigger reactive oxygen species-mediated death of human prostate cancer cells J Biol Chem 2010 285 34 26558 26569 10.1074/jbc.M109.063255 20571029 PMC2924093

[B48] Xiao D Singh SV p66Shc is indispensable for phenethyl isothiocyanate-induced apoptosis in human prostate cancer cells Cancer Res 2010 70 8 3150 3158 10.1158/0008-5472.CAN-09-4451 20354186 PMC2855757

[B49] Xiao D Singh SV Phenethyl isothiocyanate sensitizes androgen-independent human prostate cancer cells to docetaxel-induced apoptosis in vitro and in vivo Pharm Res 2010 27 4 722 731 10.1007/s11095-010-0079-9 20182772 PMC2837783

[B50] Trachootham D Zhou Y Zhang H Demizu Y Chen Z Pelicano H Chiao PJ Achanta G Arlinghaus RB Liu J Selective killing of oncogenically transformed cells through a ROS-mediated mechanism by beta-phenylethyl isothiocyanate Cancer Cell 2006 10 3 241 252 10.1016/j.ccr.2006.08.009 16959615

[B51] Badia E Oliva J Balaguer P Cavailles V Tamoxifen resistance and epigenetic modifications in breast cancer cell lines Curr Med Chem 2007 14 28 3035 3045 10.2174/092986707782794023 18220739 PMC2789301

[B52] Thomas S Thurn KT Bicaku E Marchion DC Munster PN Addition of a histone deacetylase inhibitor redirects tamoxifen-treated breast cancer cells into apoptosis, which is opposed by the induction of autophagy Breast Cancer Res Treat 2011 130 2 437 447 10.1007/s10549-011-1364-y 21298336 PMC3760725

